# Defective viral genomes: advances in understanding their generation, function, and impact on infection outcomes

**DOI:** 10.1128/mbio.00692-24

**Published:** 2024-04-03

**Authors:** Justin W. Brennan, Yan Sun

**Affiliations:** 1 Department of Microbiology and Immunology, University of Rochester Medical Center, Rochester, New York, USA; The Ohio State University, Columbus, Ohio, USA

**Keywords:** defective viral genomes, genomic sequences, disease severity, therapeutic interfering particle, interferon, interference, persistence, genomic structures

## Abstract

Defective viral genomes (DVGs) are truncated derivatives of their parental viral genomes generated during an aberrant round of viral genomic replication. Distinct classes of DVGs have been identified in most families of both positive- and negative-sense RNA viruses. Importantly, DVGs have been detected in clinical samples from virally infected individuals and an emerging body of association studies implicates DVGs in shaping the severity of disease caused by viral infections in humans. Consequently, there is growing interest in understanding the molecular mechanisms of *de novo* DVG generation, how DVGs interact with the innate immune system, and harnessing DVGs as novel therapeutics and vaccine adjuvants to attenuate viral pathogenesis. This minireview focuses on single-stranded RNA viruses (excluding retroviridae), and summarizes the current knowledge of DVG generation, the functions and diversity of DVG species, the roles DVGs play in influencing disease progression, and their application as antivirals and vaccine adjuvants.

## INTRODUCTION

Defective viral genomes (DVGs) are derivatives of their parental viral genomes containing lethal mutations, drastic truncations, or genomic rearrangements rendering them infection incompetent in the absence of a co-infecting standard virus. Certain DVGs, containing the minimum requirements for packaging and conditional replication in the presence of the standard virus, can be incorporated into virus particles to form defective interfering particles (DIPs). DIPs interfere with the replication of co-infecting standard viral genomes by competing for critical viral proteins required for viral replication (1, 2). Additionally, DVGs are well-documented triggers of the antiviral response, and roles for DVGs promoting persistent viral infection and impacting viral evolution have also been explored. The development of modern sequencing and detection methods has further implicated DVGs in modulating the course of disease caused by viral infection. Given the versatile roles of DVGs in viral pathogenesis, the molecular basis of DVG generation has received increased attention aimed at manipulating this process. This minireview focuses on two major types of DVGs and provides an overview of potential DVG generation mechanisms, the functions of DVGs/DIPs, the impact of DVGs/DIPs on inflammation and disease including COVID-19, and their applications in combating viral infections.

## GENERATION OF DVGS

**Fig 1:**
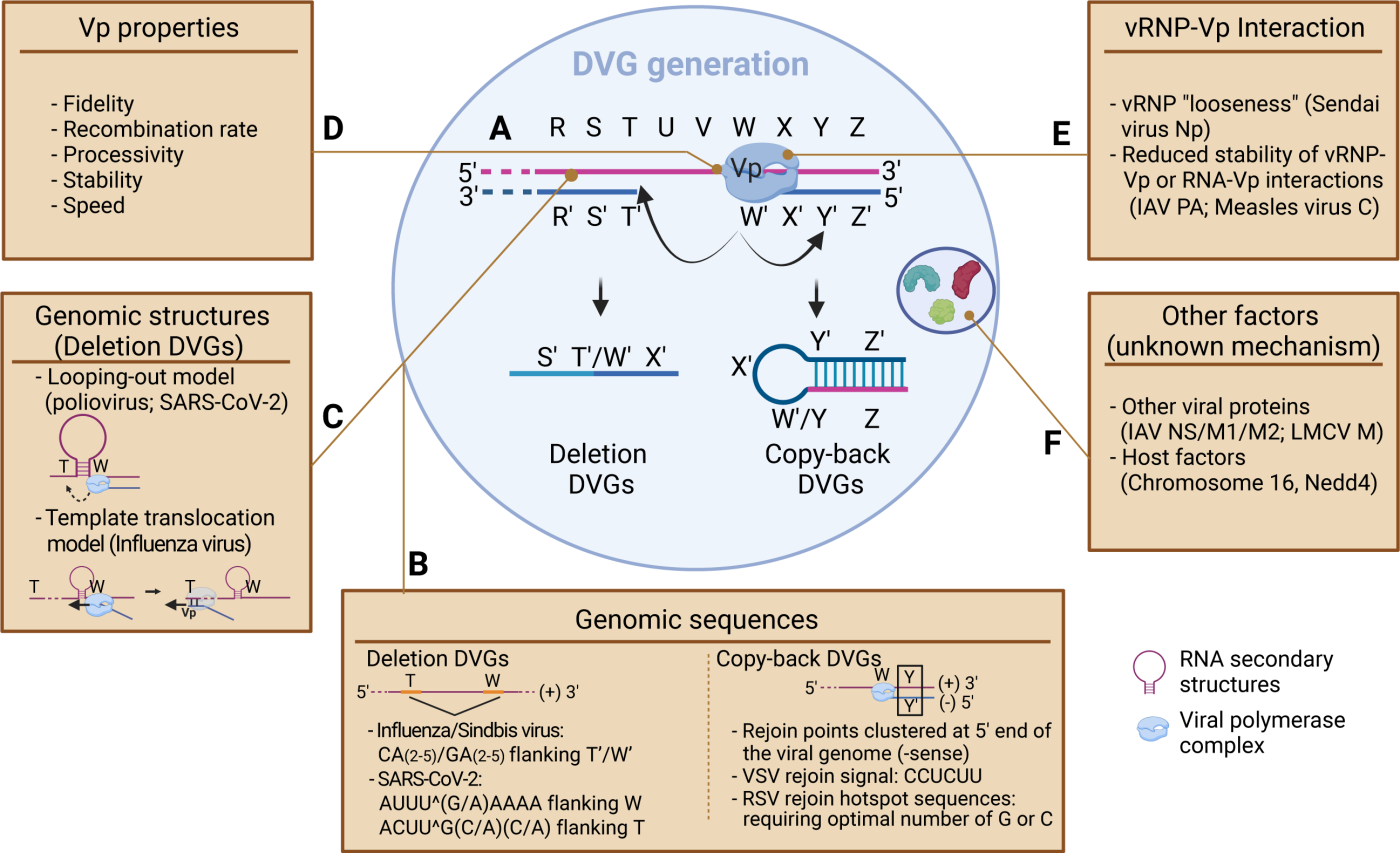
Scheme of mechanisms by which viral and host factors contribute to DVG generation. The generation of deletion and cbDVGs are schematically graphed in (**A**). Briefly, the viral polymerase complex (Vp) dissociates from the template strand (pink, +sense) at position W. Reinitiation of the progeny associated Vp at a different position, T, on the same template strand, results in the formation of a deletion DVG with breakpoint T′/W′. Vp reinitiation on the nascent strand (blue, −sense) at the position Y′, results in the formation of a cbDVG with breakpoint W′/Y. Both virus and host factors can impact this process. As listed in the boxes, viral factors include viral genomic sequences (**B**), genomic structures (**C**), Vp properties (**D**), Vp-vRNP interactions (**E**), and other viral proteins (**F**). (**B**) Specific sequences flanking DVG junction sites are documented for both deletion and copy-back DVGs. For deletion DVGs, such sequences identified from influenza, Sindbis virus, and SARS-CoV-2 DVGs are listed. ^ indicates the position of junction sites in the SARS-CoV-2 genome (W or T). For cbDVGs, the location and sequence requirements for rejoin points (Y or Y′) are documented in panel **B**. (**C**) Two models have been proposed to explain how genomic structures impact deletion DVG generation: the looping-out model and template translocation model. In looping-out model, an RNA-RNA interaction between two DVG junction sites brings two sequences spatially close to facilitate Vp movement from W to T. In the template translocation model, RNA structures formed near the dissociation position, W, cause the Vp to temporarily halt synthesis of the nascent strand. The Vp continues moving forward along the template until a hybridization event occurs between the template and nascent strands at position T. Hybridization triggers the resumption of nascent strand synthesis. (**F**) Several host factors have been documented to alter DVG generation, but most mechanisms are unknown.

Various species of DVGs have been detected in most RNA virus families (1–9), the majority of which fall within two major classes: deletion and copy-back (cbDVG). A comprehensive list of virus families and their DVGs has been previously reviewed (10). Deletion DVGs are commonly observed in both positive-sense viruses and influenza viruses and typically bear large internal truncations ablating critical gene sequences while retaining the promoter regions and *cis*-acting sequences at the genomic termini required for their replication and packaging (11, 12). Broadly, deletion DVG generation is thought to occur during viral genomic replication when the viral polymerase dissociates from and reinitiates with the same sense template at different locations in an intra- or intermolecular manner, leading to the deletion of internal sequences (Fig. 1A) (5, 13, 14). cbDVGs are prevalent among non-segmented negative-sense RNA viruses such as rhabdovirus, paramyxoviruses, pneumoviruses, and filoviruses. cbDVGs constitute rearranged genomes characterized by reverse complementary 5′ and 3′ ends and form a theoretical panhandle structure (15–17). Unlike deletion DVGs, cbDVG generation is thought to occur when the viral polymerase dissociates from the template strand and reinitiates with the nascently synthesized strand and continues elongating through the 5′ end (Fig. 1A) (18, 19). Specifically, the generation of deletion DVGs is thought to rely on a copy-choice-mediated mechanism where the viral polymerase switches from the donor template to the acceptor template while remaining bound to the nascent strand. Less is known regarding the mechanisms of cbDVG generation. In this section, we review evidence describing the roles of genomic sequences, RNA secondary structures, viral proteins, and host factors in mediating the generation of both types of DVGs (summarized in Fig. 1B through F).

### Genomic sequences

Given the inherent error-prone nature of viral RNA-dependent RNA polymerases (RdRps), it was historically accepted that DVG generation is a random process. However, the dissociation (break point) and re-initiation (rejoin point) positions of both deletion and cbDVGs are consistently observed to cluster in certain genomic regions across different virus families (8, 20–26), strongly arguing against the completely random generation theory. Additionally, microhomologies and specific nucleotide compositions have been observed to flank DVG break and rejoin points (3, 6, 27), further suggesting a sequence dependency for DVG generation (Fig. 1B). For deletion DVGs, GAA, and CAA sequences, each with 2–5 As, were observed 5′ and 3′ to influenza A virus (IAV) DVG junction sites, respectively (27). Interestingly, the exact same sequence pattern was also found in proximity to Sindbis virus deletion DVG junction sites (6). For SARS-CoV-2 and MERS-CoV, their DVGs reportedly preferred break and rejoin sites with 2–7 nts of sequence overlap. The genomic sequences upstream of the start and stop sequences resulting in DVG junctions were U enriched and depleted of A and G, while the genomic sequences downstream of the junction start and stop sequences were enriched for G and A and depleted of U (Fig. 1B). Notably, these nucleotide compositions differed from SARS-CoV-2 and MERS-CoV transcription regulatory sequence (TRS)-like sequences, respectively, together suggesting that coronaviruses use distinct sequence patterns for DVG generation (3). Consistent with this finding, recent transcriptomic analysis of SARS-CoV-2 *in vitro* infection and patient-derived samples further identified four TRS-independent genomic “hotspots-”mediating deletion DVG formation (20).

For cbDVGs, despite the lack of sequence homology flanking their break and rejoin points (18), hotspots and nucleotide requirements for their generation have been observed. RNA-sequencing of parainfluenza virus 5, Nipah virus, and Ebola virus cbDVGs recognized dominant populations of cbDVGs rejoining at the 5′ end of the genome (8, 21, 22). A C protein-deficient measles virus showed cbDVG break points distributed across the genome, with rejoin points clustering within the final 200 nucleotides of the genome (23). Moreover, *in vitro* and patient-derived respiratory syncytial virus (RSV) cbDVGs were observed to use discrete break and rejoin “hotspots” for their generation. Interestingly, compared to the wide distribution of break hotspots, RSV rejoin hotspots were clustered at the end of the L gene and within the viral trailer sequence, also spanning the last ~200 nucleotides (9). The similarity of cbDVG rejoin point selection among Ebola, parainfluenza 5, Nipah, RSV, and measles viruses hints at a conserved mechanism of cbDVG generation among members of *mononegavirales*. Examining cbDVG rejoin sequences, comparison of vesicular stomatitis virus (VSV) cbDVGs identified a CCUCUU sequence in the 5′ stem region that may act as a signal to the viral polymerase for rejoin point selection (28). Similarly, mutations of RSV rejoin hotspot sequences demonstrated that a stretch of Us abolished cbDVG formation/accumulation in the mutated region, whereas G/C mutations did not (9). These results not only suggest that an optimal number of G or C ribonucleotides is required for cbDVG rejoin point selection of RSV but also establish the possibility of genetically manipulating cbDVG populations by altering the genomic sequences of rejoin hotspots. One caveat of these studies, however, is that initial DVG generation is currently indistinguishable from their subsequent accumulation. As a consequence, changes in DVG repertoire and/or total abundance may be influenced by fitness advantages of dominant DVG species rather than reflecting the initial DVG generation pattern.

### Genomic structures

RNA secondary structures play key roles in viral replication, translation, packaging, and evasion of cellular dsRNA sensors for many positive-sense RNA viruses (29–32). Studies have experimentally or computationally identified RNA secondary structures in the genomes of different positive-sense RNA viruses contributing to their DVG generation (20, 33). For example, predicted secondary structures of the poliovirus genome correlated with recombination sites of its deletion DVGs, leading to the hypothesis of the looping-out model (Fig. 1C). In this model, an RNA structure, forming a loop, brings two distant genomic sites in close spatial proximity to facilitate viral polymerase movement between recombination sites (33). Recently, high-throughput methods probing intracellular RNA base pairing such as selective 2′ hydroxyl acylation analyzed by primer extension (SHAPE) and cross-linking of matched RNAs and deep sequencing (COMRADES) have allowed for the identification of both short- and long-range RNA interactions, especially for viral genomes that are not largely coated with viral proteins, if not completely naked (31, 34–37). Using COMRADES, Ziv et al. identified genome-wide RNA secondary structure architectures during Zika virus and SARS-CoV-2 infections *in vitro* (36, 37). We further analyzed the relationship between SARS-CoV-2 genomic secondary structures and its DVG junction positions which yielded a strong positive correlation, especially in the genomic region encoding orf7, orf8, and N genes (20). Overall, these data suggest that genomic structures are utilized by the viral polymerases of positive-sense RNA viruses to form deletion DVGs. However, identification of the responsible structures and understanding mechanistically how they mediate DVG formation remain challenging.

Compared to positive-sense RNA viruses, the genomes of negative-sense RNA viruses are better encapsidated with the nucleoprotein to form a helical viral ribonucleoprotein complex (vRNP) (38). Consequently, it was thought that the formation of RNA secondary structures and base-pairing within the genomes of these viruses was restricted by the vRNP. However, the break and rejoin regions of IAV deletion DVGs correlated with one another in adjacent regions of viral RNA in the vRNP tertiary structure. Such juxtaposition of vRNPs was suggested to facilitate the template switching of the viral polymerase to form DVGs (27). Additionally, recent evidence indicates that the nucleoprotein coverage of the influenza genomic segments is incomplete (39), which was further verified by the identification of inter- and intra-gene segment RNA structures (40–42). Two hypothetical models have been proposed to explain how intra-segment structures mechanistically contribute to the selection of DVG junction sites in influenza (43). First, the looping-out model, as previously discussed for poliovirus (33). Second, the template translocation model (Fig. 1C). In this model, an RNA structure induces the viral polymerase to pause synthesis of the nascent strand while still moving along the template until it recognizes a signal to resume nascent strand synthesis at a downstream rejoin point on the same template (43–45). This signal might be the complementarity between the break point of the nascent strand and the rejoin point on the template. For negative-sense viruses generating cbDVGs, RNA structures were reported at both ends of the RSV genome (46). Furthermore, a single mutation within the Sendai virus nucleoprotein was observed to reduce the density of vRNPs and enhance cbDVG generation. It was hypothesized that this mutation may impact the “looseness” of the vRNP, to allow for base-pairing between genomic and antigenomic vRNPs at certain regions, facilitating cbDVG generation (47). Altogether, increasing evidence supports the role of RNA secondary structures mediating deletion DVG generation. For most negative-sense viruses producing cbDVGs, structures within genomic RNAs and interactions among vRNPs have begun to emerge. This implies their potential roles in cbDVG generation by physically bringing the break and rejoin sequences into proximity or by facilitating the dissociation and re-initiation of RdRps separately.

### Roles of viral proteins

Because DVG generation occurs during an aberrant round of viral genomic replication, several studies have examined how viral RdRps, and their associated proteins, impact DVG generation. Although largely unknown, emerging evidence suggests that there is an intrinsic link between DVG production (majorly deletion DVGs) and the fidelity, processivity, speed, and stability of viral polymerases (Fig. 1D). A mutation attenuating RdRp fidelity in Sindbis virus was shown to enhance recombination and DVG generation (6). Mutations ablating nsp14 exoribonuclease (ExoN) activity in murine hepatitis virus (coronavirus) proportionately enhanced DVG amounts relative to subgenomic mRNAs (sgmRNAs, TRS-dependent) despite a significant reduction in the absolute amount of both viral RNAs (3). In this case, nsp14 mutations impaired both the proofreading (fidelity) and recombination abilities of the viral polymerase. In the same study, the authors suspected that nsp14 ExoN mutations impaired its interaction with nsp12, thus further altering the stability, speed, and processivity of the polymerase complex. Furthermore, mutations impacting polymerase-polymerase interactions reportedly alter DVG generation in poliovirus and influenza virus, wherein polymerase oligomerization is required for function (24, 48, 49). More studies are necessary to dissect the intrinsic relationship between these properties of viral polymerases and their direct roles in DVG generation.

The interaction between the viral polymerase and the vRNP has been implicated in DVG generation (Fig. 1E). Mutations R683A and D529N in the IAV PA subunit were observed to enhance and reduce DVG production, respectively. Interestingly, both mutations mapped to the PA C-terminal domain in spatial proximity of one another and were hypothesized to impact the stability of the vRNP-polymerase interaction during elongation (48, 50). Similarly, the measles virus C protein is suggested to stabilize the vRNP-polymerase interaction to reduce the frequency of premature chain termination (51) and the C protein of measles and Sendai virus was observed to attenuate cbDVG production (23, 51, 52).

Viral proteins not canonically associated with the viral polymerase have also been observed to affect DVG generation and accumulation (Fig. 1F). For example, the PPXY domain of the lymphocytic choriomeningitis mammarenavirus (LCMV) matrix protein is required for DIP formation, but not for standard virus particle formation (53). An IAV NS2 mutation was reported to extragenically dysregulate the replication and expression of the PA gene to enhance DVG generation (54). Similarly, the exchange of the NS gene segment from a highly pathogenic H5N1 strain with the cognate H1N1 NS gene segment resulted in the enhanced *de novo* generation of DVGs, underscoring the role of influenza virus reassortment in DVG production (55). IAV M1 and M2 also reportedly play a role regulating deletion DVG production through an unknown mechanism (56). Overall, the role of viral proteins influencing DVG generation is multifaceted. Polymerase functions and RNA-polymerase complex stability are implicated in DVG generation, necessitating consideration when assessing DVG synthesis. Other viral proteins are also likely contributors to both DVG generation and accumulation though future studies will be required to better understand these mechanisms.

### Host factors

Host factors have been shown to modulate the production of DVGs/DIPs (Fig. 1F). West Nile virus (WNV) DIP production was enhanced in cells derived from genetically resistant mice (57) and avian influenza virus strains were observed to produce an increased abundance of DVGs during infection in mammalian cells (58). Likewise, neural precursor cell expressed derived developmentally down-regulated protein 4 (Nedd4) family E3 ubiquitin ligases interact with the LCMV matrix protein to specifically promote DIP production (53, 59). Conversely, measles virus vaccine strains and Sendai virus did not produce detectable cbDVGs in human fetal lung fibroblast cells or in chicken embryo lung cells, respectively (60, 61). Evidence also linked human chromosome 16 to the suppression of VSV DVG generation in a human-mouse hybrid cell line via unknown mechanisms (62). Moreover, despite lacking direct evidence, variations in temperature may impact DVG/DIP production possibly by altering: the functionality of RdRps, the stability of genomic secondary structures, and innate immune responses (63). For example, temperature impacts IAV polymerase fidelity (64, 65). At 37°C, purified IAV RdRps rapidly dissociated from the RNA template, reducing its processivity (66). High temperatures destabilize RNA structures in general. A stabilized stem-loop structure in WNV attenuates genomic replication at 28°C; however, at 37°C, the flexibility of this structure is enhanced, relieving the suppression of genomic replication (67). Given the role of RNA structures in DVG generation for positive-sense RNA viruses, temperature changes may also impact DVG generation, especially for flaviviruses. Interestingly, temperature can directly impact the host response to RNA virus infections such as influenza virus (68), rhinovirus (69), and SARS-CoV-2 (70, 71), which may change the level of viral proteins available for DVG replication. Taken together, despite the current absence of described mechanisms, a growing body of data implies an indispensable role of host factors in DVG production.

## FUNCTIONS OF DVGS/DIPS

### Interference with standard virus replication

**Fig 2:**
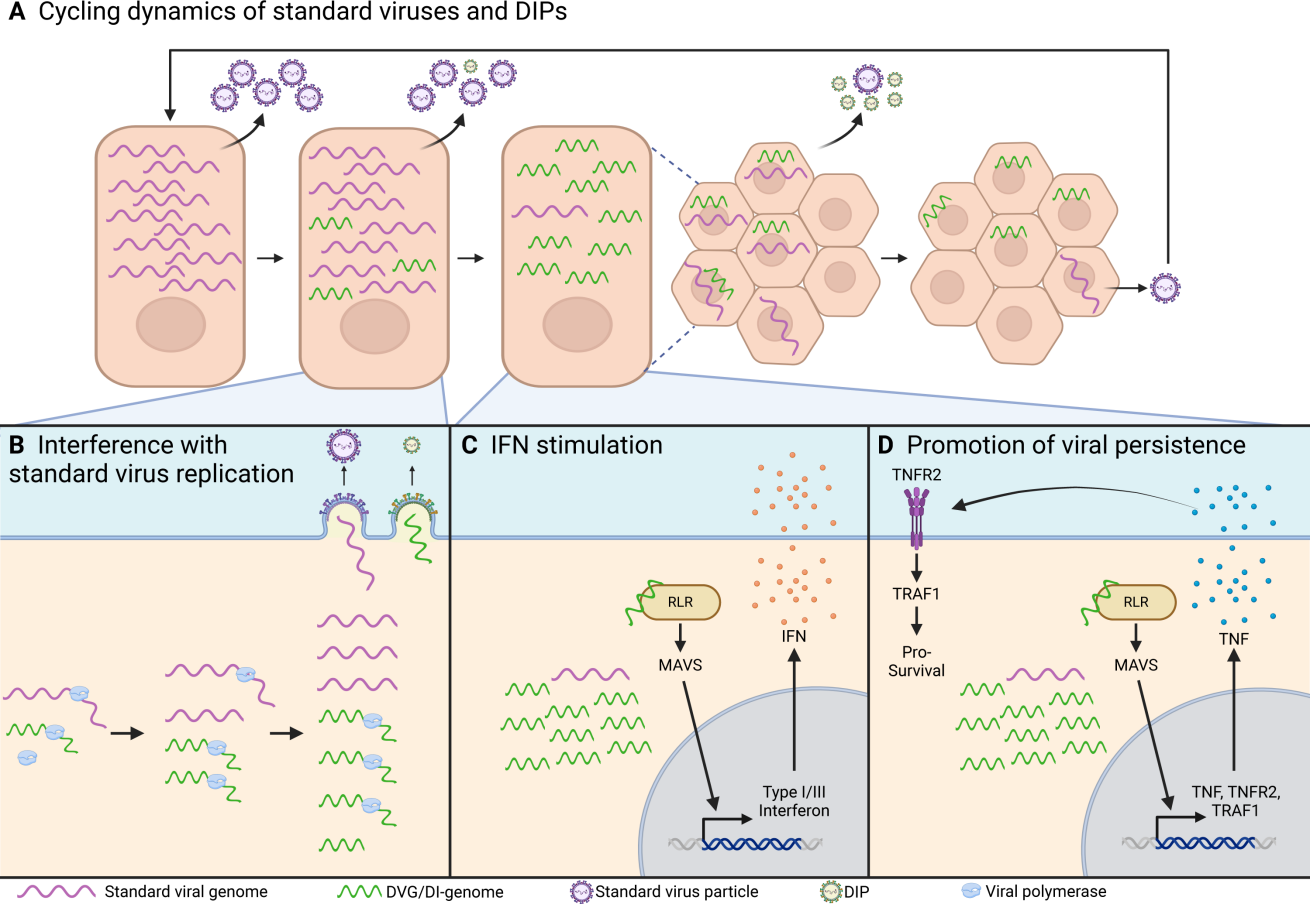
Scheme of DVG/DIP cycling dynamics and their functions. (**A**) Proposed mechanism of DIP/standard virus asynchronous cycling. A cell is first infected by the standard virus leading to the infection of additional cells. As the titer of the standard virus increases, *de novo* generation of DVGs/DIPs occurs. DIPs conditionally replicate in the presence of the standard virus thereby interfering with its replication, leading to the accumulation of DIPs and a consequent reduction of the standard virus titer. Within a population of cells, reduced standard virus titers eventually lead to a significant reduction of cells co-infected with both the standard virus and DIPs. Because DIPs are dependent on a standard “helper” virus for their replication and packaging, cells infected with only DIPs will not yield further viral progeny and DIP titers correspondingly decrease. In contrast, cells infected with only the standard virus will produce standard virus particles, leading to an increase of standard virus titers. These asynchronous oscillations in standard virus and DIP titers are referred to as the Von Magnus effect. (**B**) Interference with standard virus replication. DVGs containing the *cis*-acting elements required for genomic replication and packaging into the virus particle interfere with standard virus replication by competing for a limited number of critical viral proteins within a co-infected cell. This leads to the production and accumulation of DIPs at the expense of the standard virus. (**C**) Interferon induction. Cells enriched with DVGs, particularly cbDVGs, upregulate types I and III IFN signaling by RLR detection/activation and MAVS signaling, given that cbDVGs theoretically form the canonical RIG-I ligand. (**D**) Promotion of persistent viral infection. DVG-enriched cells upregulate TNF, TNFR2 and TRAF1 expression. TNF signaling through TNFR2/TRAF1 activates pro-survival pathway and protects DVG enriched cells from apoptosis. The few standard viral genome within DVG-enriched cells further facilitate the establishment of persistent viral infection.

First described by Von Magnus, numerous studies have recognized the ability of DVGs/DIPs to directly interfere with the production of their parental helper viruses (1, 4, 7, 72–75). Gradient centrifugation-based evidence indicates that DIPs are physically smaller than the standard viral particles due to the shorter sequence length of DVGs within DIPs (DI genomes) compared to their parental genomes (1, 76). The shorter sequence lengths of both deletion- and cbDVGs are thought to confer DI genomes with enhanced replication kinetics (2). cbDVGs, specifically, possess two trailer promoter sequences. The trailer promoter sequence of negative-sense RNA viruses producing cbDVGs largely favors genomic replication relative to the leader promoter, conferring cbDVGs an additional replicative advantage (77, 78). Likewise, DVGs within VSV DIPs have been observed to acquire mutations in the promoter sequences to increase polymerase or nucleoprotein binding for enhanced replication (79). Consequently, by occupying viral replication machinery, DI genomes limit the availability of the proteins required for replication of the standard helper virus thereby reducing the amount of standard virus produced (18, 19, 80, 81) (Fig. 2B). Additionally, certain DVGs can compete for critical viral structural proteins required for packaging. For example, deletion DVGs of IAV were observed to exhibit increased segment-specific packaging efficiency via preferential incorporation into virus particles over the full-length segment they were derived from (82–84). Competition for incorporation into the virus particle, however, is not invariably true for all DVGs. Specifically, DVGs lacking *cis*-acting sequences required for packaging may be inefficiently packaged or completely fail to do so. As demonstrated by high-throughput random deletion library sequencing (RanDel-seq) of Zika virus variants, genomes baring deletions in the *cis*-acting elements at the 5′ and 3′ UTRs were not tolerated during passaging (85). This principle also applied to SARS-CoV-2 *cis* elements (86). Furthermore, a deletion DVG from a naturally selected SARS-CoV-2 DIP expressed a nsp1-10 fusion protein, which further attenuated the helper virus via a currently unknown mechanism (75). IAV deletion DVGs have also been observed to express polypeptides. However, these DVG-encoded proteins did not exhibit attenuation activity (87). While DIPs interfere with standard virus replication, they also compete with one another leading to the selection and accumulation of the fittest DIP species given that the generation and accumulation of DIPs requires multiple passages at a high MOI. For example, the rule of six stipulates that paramyxovirus polymerases efficiently replicate viral genomes whose lengths are only a multiple of six nucleotides (88). Correspondingly, the vast majority of cloned measles virus cbDVGs obeyed the rule of six, likely due to their replicative advantage over those that did not (23, 89). Likewise, an artificial IAV deletion DVG library was generated to study the impact of various deletion lengths and DVG segment origins on DVG replication. The use of this library recognized that certain DVG species were lost during infection thereby influencing the observed deletion species in the final population (90).

### Interferon induction

Both deletion and cbDVGs stimulate interferon (IFN) responses (Fig. 2C). cbDVGs are potent agonists of the types I and III IFN responses, via RIG-I-like receptor (RLR) activation, and inducers of pro-inflammatory cytokines IL-6, tumor necrosis factor (TNF), and IL-1β (91–98). The innate immune response activated by cbDVGs further enhances antigen presentation and dendritic cell maturation (96, 97, 99). cbDVGs from Sendai virus more efficiently induced innate IFN responses compared to their homologous full-length viral genomes (91, 96, 97). Because cbDVGs theoretically form a blunt-ended dsRNA stem motif with a di/triphosphorylated 5′ end lacking 2-O methylation and a 5′-methylguanosine cap, cbDVGs theoretically form the canonical RIG-I ligand, which is not present in standard viral genomes. In support, cbDVGs from measles and Sendai virus have been shown to interact with RIG-I to induce IFNβ expression (100–102). The 5′-di/triphosphate dsRNA motif of *in vitro* transcribed measles virus cbDVG RNA is required for IFNβ induction (103). RSV cbDVGs have been observed to promote a MAVS-mediated IFN response and were further observed to strongly induce IFNλ1 expression associated with IRF1 activation independent of IFN signaling (92). Interestingly, phosphatase treatment of *in vitro* transcribed cbDVGs largely attenuated, but did not totally ablate, their capacity to induce IFN expression (99, 100). This suggests that there are other RNA motif(s) within cbDVG RNAs that can be detected by RIG-I to stimulate IFN. Indeed, a major Sendai virus cbDVG, DVG-546, forms a predicted RNA secondary structure spanning the break/rejoin junction sequence that promotes binding to RIG-I (100). It is important to note that while cbDVGs theoretically can form a blunt-ended dsRNA motif and other RNA secondary structures, this assumes the absence of the viral nucleoprotein which, during infection, encapsidates cbDVGs. As such, it was shown that the encapsidation of measles virus cbDVGs attenuated their immunostimulatory capacity, likely by disrupting the formation of the dsRNA stem motif or another immunogenic RNA secondary structure (89). Unlike RIG-I, MDA5 is thought to recognize long spans of dsRNA and complex RNA secondary structures in a phosphate-independent manner (104). The overall role of MDA5 in cbDVG-mediated IFN responses is controversial. For example, MDA5 was observed to play a key role in the early detection of Sendai virus cbDVGs in dendritic cells, enhancing their maturation (96), and a specific Sendai virus cbDVG motif reportedly enhanced both RIG-I and MDA5 binding via an electrophoretic mobility shift assays (100). Conversely, cbDVGs generated from C or V protein-deficient measles viruses reportedly interacted with RIG-I but not MDA5 (89).

Deletion DVGs identified from dengue (*Flaviviridae*), Sindbis (*Togaviridae*), and influenza virus (*Orthomyxovidae*) DIPs all showed potent IFN stimulation (105–107). One engineered deletion DVG from poliovirus (*Picornaviridae*) also induced strong IFN responses (108). DVGs from chikungunya (*Togaviridae*) virus were reported to have antiviral effects, potentially via both interference and immuno-stimulation activities (109). We recently reported a strong positive correlation between DVG amount and IFN responses during SARS-CoV-2 (*Coronaviridae*) infection (20). It is thought that either deletion DVGs themselves (101, 110) or double-stranded RNA (dsRNA) intermediates (10, 111, 112) generated during the replication of DVGs activate pattern recognition receptors, such as RLRs, to trigger IFN pathways inducing the expression of IFNs and IFN-stimulated genes (ISGs). However, the specific sequences or putative secondary structures that RLRs may be recognizing in deletion DVGs are unknown. Note, the DVGs in this section can be packaged to viral particles, forming DIPs.

Evidence supports that DVGs activate pathways inducing IFN expression even in the presence of viral proteins antagonizing these same pathways. This raises the question: how do DVGs overcome IFN antagonism to induce robust IFN responses? Within the single infection, subpopulations of cells have been observed to disproportionally accumulate either the standard virus or DVGs, resulting in distinct transcriptomic profiles and cell fates (113–115). RNA fluorescent *in situ* hybridization was used to identify heterogeneity among cells enriched with cbDVGs or standard virus during RSV and Sendai virus infection *in vitro* (113). It was found that cells enriched with Sendai virus cbDVGs: exhibited less standard virus replication and thus expressed fewer virally encoded IFN antagonists, poorly interacted with cellular trafficking components, and released few virus particles. Due to the large amount of stimuli (cbDVGs) and the relatively low amount of IFN antagonists, these DVG-enriched cells strongly expressed genes associated with the innate immune response. In contrast, cells enriched with standard viral genomes produced both infectious virus particles and defective virus particles (115) but expressed few IFN-related genes (113). Similarly, single-cell RNA sequencing of IAV-infected cells revealed that cells with a high content of DVG reads also highly expressed genes associated with the IFN response and exhibited a corresponding reduction of viral transcripts (114). This segregation of IFN-producing cells and virus particle-producing cells, although requiring verification *in vivo*, may be one of the mechanisms allowing DVGs to circumvent the well-established blockage of IFN pathways by viral proteins.

### Promotion of persistent viral infection

DIPs produced by positive- and negative-sense viruses during high MOI infections have been observed to promote persistent infection in tissue culture (7, 116–118). It has been described, *in vitro*, that DIPs and standard viruses asynchronously cycle during persistent infection (119, 120). At the population level, Huang and Baltimore proposed that during infection, the high titer of the standard virus leads to the production and accumulation of DIPs which, in turn, interfere with the standard virus leading to DIP predominance over the standard virus. Because DIP replication is dependent on a co-infecting helper virus, low standard virus titers will reduce the cell population that co-infected with both standard virus and DIP and decrease the amount of DVGs in co-infected cells. With few DIPs and standard virus particles remaining, two scenarios may arise: cells will be infected with either only DIPs, yielding a non-productive infection, or with only the standard virus, giving an increase in the concentration of the standard virus and repeating this cycle. The asynchronous cycling of DIP and standard virus titers is known as the Von Magnus effect (1, 2) (Fig. 2A). At the molecular level, the two subpopulations of DVG-enriched and standard virus (full-length viral genome)-enriched cells also play a role in establishing the persistent infection. During Sendai virus infection *in vitro*, it was shown that cells enriched with standard virus died of TNF-mediated apoptosis, while cells enriched with DVGs upregulated the expression of TNF receptor 2/receptor-associated factor 1 (TNFR2/TRAF1) via RLR signaling. TNFR2/TRAF1 activated pro-survival pathways to protect DVG-enriched cells from apoptosis (Fig. 2D). In DVG-enriched cells, small amounts of full-length viral genomes were also present, producing the viral machinery necessary for DVG replication. The longer longevity of DVG-enriched cells promoted persistent infection *in vitro* (113). Of course, other mechanisms may be utilized by DVGs to facilitate viral persistence in other viruses. In insects, for example, Sindbis virus deletion DVGs serve as templates for viral DNA synthesis, in a dicer-2-dependent manner, which enhances fly survival and thereby promotes persistent infection (121). One limitation is that most of these studies were restricted to tissue culture because most RNA viruses are thought to only cause acute infection *in vivo* and in humans. In RNA viruses that can establish persistent infection *in vivo*, such as LCMV, DVGs were detected in the long term (122, 123). As detection methods have advanced, a growing number of RNA viruses such as Ebola, RSV, human metapneumovirus, Zika, and chikungunya virus have been observed to persistently shed viral RNAs, proteins, and/or virus particles in humans (124–129). With these observations, DVG-mediated persistent infection and how it relates to human disease has gained significant attention. This will be further discussed in the following section. To minimize prolonged viral infection, more studies are needed to understand the mechanisms of DVG-promoted viral persistence on a virus-specific basis.

### Evolution

Emerging evidence suggests that DVGs may contribute to the fitness of the standard virus. Given the low-fidelity of viral RdRps and the high rates of recombination among positive-sense RNA viruses, raises the question of whether DVG generation provides a selective advantage for their standard viruses and whether standard viruses, in turn, regulate their generation of DVG populations as means of adapting to new environments/hosts. For example, SARS-CoV-2 is a highly recombinogenic virus reportedly 10 times more recombinogenic than MERS-CoV (3). This high degree of recombination is thought to contribute to the rapid emergence of novel SARS-CoV-2 variants and, at the same time, this high degree of recombination in SARS-CoV-2 has been correlated with DVG generation (3, 20). Additionally, it has been proposed that DVGs may provide a pool of viral sequences, potentially containing adaptive mutations, that could be incorporated into the standard viral genome via recombination to enhance standard virus fitness (10). Lastly, given the immunostimulatory and interference activities of DVGs, it is possible that DVGs apply a selective pressure to select for the standard viruses with increased replication kinetics and better antagonism of the host innate immune response. These hypotheses, however, remain speculative currently and require thorough assessment.

## THE ROLE OF DVGS/DIPS IN INFLAMMATION AND DISEASE

While DVGs derived from RSV, influenza, dengue, and measles virus have been detected during infection in humans (4, 25, 92, 130, 131), studies investigating the impact of DVGs on infection outcomes have only recently begun to emerge. Multiple contemporary studies have identified correlative roles for DVGs influencing disease severity in humans and have further characterized the functional roles of DVG infection in mouse models. For example, Sendai virus cbDVGs enhanced IFNβ expression, attenuated viral loads, and reduced lung pathology in mouse models (91). Likewise, in mice infected with RSV, cbDVGs rapidly induced IFNβ expression and reduced cellular, primarily neutrophil, infiltration into the alveolus. Mice infected with low cbDVG content stocks of RSV, however, had enhanced myeloid cell infiltration into the lung and correspondingly showed increased expression of the pro-inflammatory cytokine genes *IL-6*, *TNF*, and *IL-1*β with increased lung pathology (92). For deletion DVGs, mice infected with IAV stocks with high DVG/DIP content also showed a rapid induction of the type I IFN response and reduced titers of the standard virus in the lungs (91, 132). In humans, analysis of nasopharyngeal aspirates of pediatric patients infected with RSV observed that samples with detectable levels of cbDVGs showed enhanced expression of the ISGs: IFNA4, IFIT1, and RSAD2 (92). In a longitudinal cohort study wherein healthy adult participants were experimentally infected with RSV and assessed daily up to 2 weeks post-infection, those who had early detection of cbDVGs (before day 6 post-infection) had lower viral loads and reduced symptom severity scores. Whereas individuals who had delayed and prolonged detection of cbDVGs showed high viral loads and worse symptom severity scores overall. It is likely that the prolonged presence of DVGs further exacerbated inflammation already driven by high viral loads and correspondingly increased tissue damage (130). Together, these data suggest a critical role of the kinetics of cbDVG generation impacting host responses and consequently infection outcome. For deletion DVGs in humans, deep-sequencing of respiratory samples from individuals with severe IAV infection identified low abundances of deletion-DVGs relative to the standard virus genomes compared to individuals with mild infections. *In vitro* analysis of a recombinant IAV bearing a PA D529N mutation, identified from a fatal infection in humans, confirmed that the PA D529N mutant virus had a reduced accumulation of DVGs. PA D529N was also associated with the increased pathogenicity in mice, including increased lung virus titers, neutrophil infiltration, and depletion of alveolar macrophage. In contrast, a non-pathogenic virus bearing a PB2 A221T mutation, identified from mild patient infections, accumulated high levels of DVGs *in vitro* and had reduced pathogenicity in mice (48). For COVID-19, we observed more DVGs in symptomatic patients than asymptomatic patients (20), also implying the potential role of DVGs in symptom development of COVID-19. Overall, the abundance of DVGs, their generation kinetics, and duration during infection play key roles impacting disease severity.

In addition to impacting acute infection outcomes, DVGs are hypothesized to contribute to persistent infection in humans. For example, Ebola virus is documented to persist as a latent infection for months after the resolution of the acute infection (133). Correspondingly, an asymptomatic persistently infected individual was observed to sexually transmit infectious Ebola virus (133). cbDVG-specific RT-PCR-based methods and deep sequencing have detected Ebola virus cbDVGs in the testes of experimentally infected rhesus macaques (22), suggesting that cbDVGs may promote persistent infection in testes (134). Additional evidence reports that brain cells from individuals who died of subacute sclerosing panencephalitis a disease caused by a persistent measles virus infection, contained cbDVGs (131). Likewise, we detected large amounts of DVGs in one immunosuppressed COVID-19 patient with persistent SARS-CoV-2 infection (20). A confounding factor in these reports, however, is that DVGs were detected in either immune-privileged sites, such as the testes and central nervous system, or immunocompromised individuals. It is currently difficult to distinguish between a role for DVGs in directly promoting persistent infection versus viruses first establishing persistent infection followed by DVG generation. Thus, evidence providing a clear functional link between DVGs to persistent infection in humans is needed.

## APPLICATIONS OF DVGS/DIPS IN PREVENTING VIRAL PATHOGENICITY

Given their immunostimulatory activities, DVGs have been utilized as vaccine adjuvants to mitigate and prevent diseases caused by viral infection. The Sabin poliovirus vaccine, while not a DVG itself, reduced the morbidity of influenza virus infection (135). Interestingly, DVGs have been detected in the live attenuated vaccines of poliovirus, measles, and influenza virus (136–138), suggesting that additional virus-like species in these vaccines, such as DVGs, activate the innate immune response to provide protection against heterologous infection. Consequently, it is hypothesized that DVGs can act as adjuvants to enhance the immunogenicity of both live attenuated and inactivated vaccines. To test this, supplementation of an inactivated H1N1 2009 pandemic IAV vaccine with a prominent Sendai virus cbDVG enhanced the production of anti-hemagglutinin-specific antibodies (139). Likewise, the addition of a Sendai virus DVG-derived RNA oligonucleotide to an inactivated IAV vaccine strongly induced IgG2c antibody production, Th1 CD4^+^ and CD8^+^ T-cell responses against influenza virus, in a type I IFN-dependent manner, and protected against heterologous subtype IAV challenge in mice (140).

In addition to adjuvants, DIPs/DVGs have been tested as antivirals against influenza virus (141–145). DI244, a DIP derived from IAV/PR/8/34 containing one internal deletion in PB2, is well-documented to have strong antiviral effects *in vivo* and protect animal models from various influenza virus subtypes (146–149). The protection conferred by DI244 likely arises from interference, as demonstrated *in vitro*, and interferon stimulation, as protection was lost in myxovirus resistance 1 (Mx1)-deficient mice (150). Furthermore, DI244 protected mice from lethal pneumonia virus of mice heterologous challenge (151). DI244 additionally suppressed SARS-CoV-2 replication in Calu-3 cells via an IFN-dependent mechanism (152).

First proposed by the Weinberger group, therapeutic interfering particles (TIPs) are defined as “minimal versions of the pathogen engineered to replicate conditionally in the presence of the wildtype pathogen, transmit among individuals, and encode therapeutic elements that target key host or viral processes” (153). This group later stipulated an additional criterion: TIPs propagate with a basic reproductive ratio (*R*_0_) greater than one, meaning that a single TIP-infected cell results in the infection of more than one cell with a TIP in the presence of its cognate virus. Using this principle, this group engineered two SARS-CoV-2 deletion DVGs containing different lengths of the 5′ and 3′ genomic termini and further packaged them into virus-like particles or lipid nanoparticles to formulate TIPs. In hamsters, these TIPs: diminished SARS-CoV-2 titer 100-fold in the lungs, mobilized with *R*_0_ > 1 and were associated with reduced pro-inflammatory cytokine expression and less pulmonary edema (86). Furthermore, TIP-treated animals exhibited a significant reduction in virus shedding across various SARS-CoV-2 variants of concerns. This resulted in lower viral transmission to co-housed animals, with no evidence of TIP transmission (154). Importantly, these TIPs blunted the interferon response to the virus, acting majorly through interference. It is thought that these TIPs bind the conserved SARS-CoV-2 RdRp and, thus, presented a high barrier for viral development of TIP resistance.

DIPs used as therapeutic particles or TIPs have been described for Nipah (155), Zika (156), chikungunya (109), and poliovirus (108), recently. Despite *R*_0_ values were not established for these DIPs, they all efficiently suppressed virus infection or transmission in animal models. Specifically, Nipah virus DIPs protected hamsters from lethal Nipah virus challenge predominantly by interfering with Nipah virus replication (155). Similarly, a Zika DIP identified from natural infections did not upregulate IFN-related genes and exhibited strong antiviral effects against Zika virus in both mammalian and mosquito hosts, reducing transmission in the latter up to 90% (156). The packaged DVG partially lacked PrM and NS1 yet preserved the topology of the remaining viral polyprotein and *cis*-acting elements, supporting the previous RanDel-seq data identifying tolerable deletion regions within the Zika virus genome (85). In contrast, an engineered poliovirus DVG lacking the capsid-coding region induced strong innate responses and showed broad antiviral activity against SARS-CoV-2, influenza, and poliovirus *in vivo* when administered between 48 h prior and 24 h post viral infection. Excitingly, this DVG also promoted long-term protection (108).

One limitation of TIPs/DIPs as antivirals is their relatively short window for administration post infection. Currently, application of TIPs more than 2 days post infection in animal models significantly dampens their therapeutic efficacy. However, patients with viral infections frequently do not recognize symptom onset until 3–5 days post exposure, if not longer. This leaves a narrow window for TIP treatment in patients with ongoing infections. Nevertheless, TIPs are an exciting class of novel therapeutics that take advantage of the fundamental biology of DVGs/DIPs to attenuate virus replication, limit disease morbidity and viral transmission, and potentially promote long-term protective immunity.

## CONCLUDING REMARKS

Recent years have seen a renaissance of interest and research into DVGs. As novel methodologies have emerged, the field has begun to appreciate the diversity of DVG populations, their generation dynamics, functions, critical impact on human infection, and ultimately the efficacy of DVG-based therapeutics. However, numerous questions remain: what are the precise molecular mechanisms facilitating the generation of deletion- or copy back-DVGs, do viruses promote the generation of specific DVG populations with pro-viral functions, what features confer a DVG with potent immune-stimulatory or interference activities, what host/viral factors promote the accumulation of dominant DVG species, and how do these dominant species influence disease outcomes and viral evolution? Finally, can we manipulate DVG populations and/or their generation patterns to mitigate disease caused by viral infection? Despite these unknowns, DVGs represent a novel class of promising antiviral therapeutics. As these fundamental questions regarding DVG biology are answered, their translational application to enhance efficacy in mitigating severe human disease can be optimized.
